# Management of anticoagulation and factor XIII replacement in a patient with severe factor XIII deficiency and recurrent venous thromboembolic disease: case report and review of literature

**DOI:** 10.1016/j.rpth.2024.102371

**Published:** 2024-03-15

**Authors:** Laura Bounaix, Verena Schroeder, Pierre Fontana, Alessandro Casini

**Affiliations:** 1 Division of Angiology and Haemostasis, Geneva University Hospitals and Faculty of Medicine, Geneva, Switzerland; 2 Experimental Haemostasis Group, Department for BioMedical Research (DBMR), University of Bern, Bern, Switzerland

**Keywords:** anticoagulation, congenital deficiency, factor XIII, thrombosis

## Abstract

**Background:**

Thrombotic events in congenital factor (F)XIII deficiency are extremely rare. To our knowledge, we describe the first case of severe congenital FXIII deficiency associated with recurrent venous thrombotic events.

**Key Clinician Question:**

How to deal with anticoagulation treatment in patients with severe FXIII deficiency?

**Clinical Approach:**

The patient was treated with rivaroxaban and plasma-derived FXIII substitution therapy as prophylaxis without bleeding complications. We aimed at FXIII trough levels of 50% during the loading doses of rivaroxaban, then 30% during the maintenance dose of rivaroxaban, and finally 20% during the long-term use of prophylactic dose of rivaroxaban.

**Conclusion:**

Treatment of thrombotic events with rivaroxaban in patients with severe bleeding disorders seems to be safe, requiring an adaptation of the intensity of the replacement therapy.

## Essentials

• Thrombotic events in congenital factor XIII (FXIII) deficiency are extremely rare- Anticoagulation in patient with a rare bleeding disorder is a challenging clinical situation.
• We adapted the prophylacitc FXIII replacement according to the intensity of anticoagulation.
• Rivaroxaban seems safe for treating and preventing venous thromboembolism in patients with FXIII deficiency.

## 1 Introduction

Congenital factor (F)XIII deficiency is a rare recessive autosomal disease, more frequent in areas with a high rate of consanguinity [1]. FXIII plays a crucial role in hemostasis. It stabilizes the fibrin clot by α- and γ-chain cross-linking, regulates fibrinolysis by incorporating antifibrinolytic proteins, and contributes to clot size and stability by retaining red blood cells in the fibrin clot [2]. The diagnosis of FXIII deficiency is challenging as clotting tests such as activated partial thromboplastin time and prothrombin time are not affected by FXIII deficiency.

The clinical feature of FXIII deficiency is characterized by a severe bleeding phenotype, often starting in the neonatal period with bleeding from the umbilical stump and sometimes life-threatening hemorrhage including cerebral hemorrhages [3]. Thromboembolism events are extremely rare in this population. Management of thrombosis in patients with FXIII deficiency, as for any severe bleeding disease, is particularly challenging as clinicians must deal with both the bleeding and thrombosis risk. We report the case of a patient with severe congenital FXIII deficiency who suffered from recurrent thrombotic events, focusing on the strategy of anticoagulation combined with prophylaxis by plasma-derived FXIII.

## 2 Case Report

A woman, born in 1964, was diagnosed with a severe FXIII deficiency (compound heterozygous for p.His716Arg mutation in exon 15 and p.Arg260Cys mutation in exon 6 of *F13* [4]) at the age of 5 years during investigation of cutaneous hematomas (posttraumatic facial hematoma with delayed healing, hematoma of the right buttock, and a posttraumatic hematoma of the right leg and foot). She had no familial history of bleeding, and there was no consanguinity in her family. Initially, she was treated with cryoprecipitate on demand (for recurrent cutaneous hematomas).

In 1985, she was diagnosed with her first deep vein thrombosis (DVT, left popliteal vein). Cryoprecipitate infusion (no data are available on timing of the last infusion before thrombosis or posology), oral contraception, and long-haul travel were identified as thrombotic risk factors. The oral contraceptive was discontinued after this event. We do not have any further details on the duration of the anticoagulant treatment of this first thrombotic event or on the concomitant FXIII administration. The familial history of venous thrombosis was unremarkable. Ten years later, the thrombophilia workup revealed a heterozygous Leiden polymorphism of the *F5* gene. Since 1990, the patient was switched to prophylaxis (cryoprecipitate, then plasma-derived FXIII, Fibrogammin, CSL Behring). Unfortunately, we do not have any details on the initial posology and frequency of prophylaxis. Since the 2000s, she received Fibrogammin between 1750 and 2500 IU (25-35 IU/kg every 6 weeks), aiming for a trough level of >5%. No major bleeding episodes were reported.

In August 2012, she presented with a second DVT in the right femoropopliteal vein following a long-haul flight without other risk factors identified. The last injection of Fibrogammin (1750 IU, 25 UI/kg) was administered 1 week before. On the day of thrombosis, the FXIII activity was measured at 22.3% (Berichrom assay, Siemens Healthineers, on a BCS Siemens coagulometer). Thrombophilia investigations ruled out the presence of antiphospholipid antibodies, coagulation inhibitor deficiency, or FII gene polymorphism. She started rivaroxaban 15 mg twice a day for 3 weeks, followed by 20 mg per day for 6 months. During anticoagulation, the Fibrogammin injection was adapted to targeting a FXIII trough level of about 40% (35 IU/kg every 10 days). She had no bleeding complications during the anticoagulation. Even though a long-term anticoagulation could have been considered at that time, in view of the patient’s bleeding risk it was preferred to stop the anticoagulation. At the end of the anticoagulation, Fibrogammin doses were reduced, targeting a trough level of 20% of FXIII (10 IU/kg/4 weeks). A pulmonary computed tomography scan was performed 7 months after the DVT because of pulmonary infection, and chronic pulmonary embolisms were discovered without signs of chronic thromboembolic pulmonary hypertension.

In February 2022, the patient presented with a third DVT in the right femoral vein with pulmonary embolism (right lobar embolisms) without any additional identified risk factor. The last injection of Fibrogammin was 2 weeks before, with a FXIII level of 10.3% on the day of the thrombotic event. We started rivaroxaban 15 mg twice a day for 3 weeks and increased FXIII infusions to 1750 UI/kg (25 UI/kg) once a week, targeting a trough FXIII level of about 50%. After 21 days, we switched to rivaroxaban 20 mg/d for 3 months and reduced Fibrogammin infusion to 1750 UI/kg (25 UI/kg) every 2 weeks, targeting a trough FXIII level of 30%. Finally, we decided to continue long-term anticoagulation with a reduced dose of rivaroxaban of 10 mg/d and FXIII substitution every 3 weeks (1750 UI/kg) to maintain a trough FXIII level >20% (Figure). The patient never experienced any hemorrhagic event during anticoagulation till date.

**Figure:**
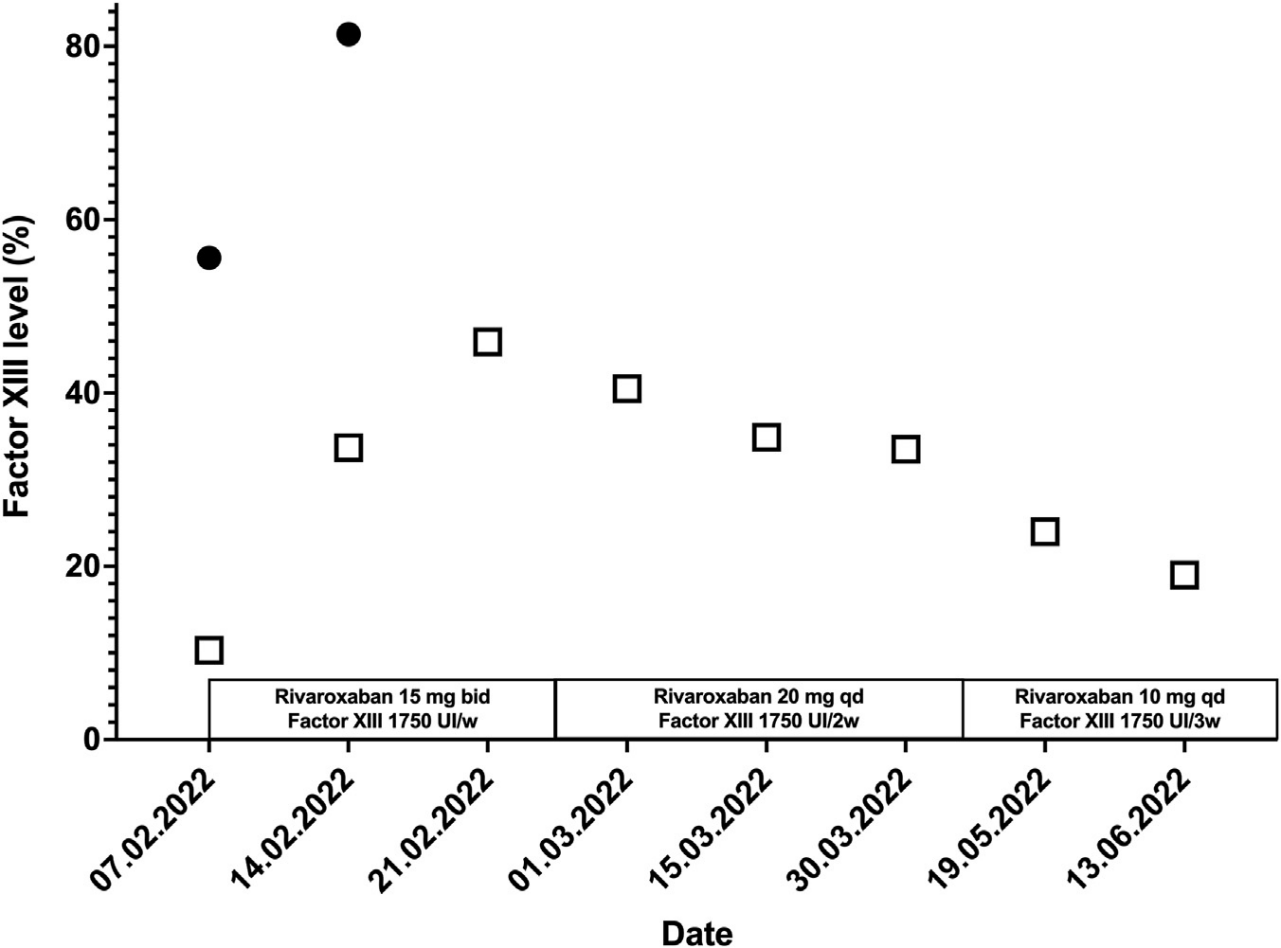
Factor (F)XIII levels during the third thromboembolic event. The empty squares correspond to trough FXIII levels. Black circles correspond to peak FXIII levels. bid, twice a day; qd, every day.

## 3 Discussion

In a mouse model of thrombosis with complete FXIII deficiency, venous thrombosis and a higher risk of pulmonary embolism in comparison with F13+/+ mouse were demonstrated, suggesting that FXIII deficiency could increase embolization risk [5]. More recently, mice in which fibrin γ-chain cross-linking by activated FXIII was eliminated were more prone to embolize their clot without modification of clot size or lysis [6], confirming that the γ-chain cross-linking and FXIII have a pivotal role in clot stability. Nevertheless, thrombotic events in severe FXIII deficiency are extremely rare. To our knowledge, only a few cases have been reported in acquired FXIII deficiency [[7], [8], [9]], and only 2 in hereditary FXIII deficiency. Almeida et al. [10] described a 3-year-old girl hospitalized for cerebral bleeding who experienced an extensive DVT following a femoral line treated by low-molecular-weight heparin complicated by oozing from the cranial wound site that eventually led to the diagnosis of FXIII deficiency. Szántó et al. [11] reported the case of a 69-year-old man with several cardiovascular risk factors and severe FXIII deficiency on FXIII prophylaxis (Fibrogammin 15 UI/kg/3 weeks), which was reduced to 9 UI/kg every 3 weeks because of unstable angina with significant coronary stenosis treated by percutaneous transluminal coronary angioplasty and clopidogrel for 3 months. Two years later, he presented an acute myocardial infarction treated by stenting, 1 dose of clopidogrel (600 mg), tirofiban, enoxaparine (0.85 mg/kg), and increased FXIII substitution (15 UI/kg, 1250 UI) [11]. After the acute phase, he was treated with clopidogrel 75 mg per day and FXIII substitution every 4 weeks (6 UI/kg, 500 UI) with a target FXIII between 15% and 24%.

Thrombotic events occasionally occur in rare congenital bleeding disorders, highlighting the complexity of the hemostatic balance [12]. Procoagulant polymorphisms such as the FV Leiden could switch the hemostatic balance in patients with a tendency to bleed. On one hand, the bleeding phenotype of patients with a bleeding disorder may be modulated by procoagulant biological factors, resulting in attenuation of clinical symptoms (ie, reduced factor concentrate consumption, bleeding episodes, and joint damage) [13]. On the other hand, procoagulant biological factors may overcome the bleeding tendency and even increase the risk of thrombotic complications, especially when other concomitant procoagulant factors are present, such as coagulation factor replacement therapy. In our case, we can speculate that the Leiden polymorphism of the FV gene has contributed to the overall procoagulant pattern of our patient. However, Leiden polymorphism of the FV gene is not associated with recurrence of thrombosis [14].

Compared with vitamin K antagonists, direct oral anticoagulants (DOACs) provide better protection against stroke or thromboembolisms while reducing the risk of major bleeding, including intracranial cerebral bleeding. Several cases of congenital fibrinogen disorders (a rare bleeding disorder sometimes associated with an increased thrombotic risk) have been treated with DOACs [15]. An EHA-ISTH-EAHAD-ESO Clinical Practice Guidance has recently been published and suggests using DOACs for stroke prevention in people with hemophilia (with FVIII or FIX >20%) presenting an atrial fibrillation [16]. To our knowledge, this is the first case of a patient with severe FXIII deficiency and recurrent venous thromboembolism treated by rivaroxaban. Even though there are no data from randomized clinical trials suggesting that the rivaroxaban concentration is correlated with a bleeding risk, recent observational studies have reported higher incidence of major bleeding according to the DOAC trough level [17]. In addition, biological studies have demonstrated that the concentration of rivaroxaban is inversely correlated with the thrombin generation [18]. Based on these clinical and biological observations, we decided to adjust the target FXIII trough level to the rivaroxaban posology. Thus, we decided to maintain a FXIII trough level above 20% during administration of the reduced dose of rivaroxaban because of the higher probability of spontaneous major bleeding complications when FXIII activity levels fall below 15 IU/dL (in patients without anticoagulation) [3].

Laboratory measurement of FXIII for diagnosis of FXIII deficiency or monitoring of FXIII replacement therapy is not trivial and can entail several pitfalls. In particular, in the low activity range below 30%, care must be taken not to overestimate FXIII activity, and confirmation with an antigen assay may be helpful. In our laboratory, we use the Berichrom assay on an Atellica COAG 360 coagulometer, Siemens Healthineers. This test is well-standardized but is known to overestimate FXIII activity, specifically in low activity range [19], unless a plasma blank without FXIII activation is subtracted [20]. Comparing our results with a biotin incorporation assay for FXIII activity measurement and an enzyme-linked immunosorbent assay for FXIII-A subunit antigen, we found a discrepancy between the 3 tests particularly when the FXIII was under 15% (FXIII activity Berichrom, 6.7%; FXIII activity “biotin incorporation assay,” 4.3%; FXIII antigen, 1.4%). More information on FXIII assays and recommendations for the diagnostic workup can be found here [21,22].

Difficulty in measuring FXIII challenged the treatment adaptation in our patient. However, despite overestimation of FXIII activity, the patient had no bleeding complication under the actual therapy.

In conclusion, we report the first case of severe congenital FXIII deficiency with recurrent thromboembolism, highlighting the difficulties of dealing with both bleeding and thrombotic risk. DOACs seem safe for treating and preventing venous thromboembolism in patients with FXIII deficiency. Prophylactic FXIII replacement therapy should be adapted to the intensity of anticoagulation. Our strategy of keeping the FXIII plasma level higher than 50% with the rivaroxaban loading dose (15 mg twice a day), >30% with the maintenance dose (20 mg every day), and >20% with the prophylactic dose of rivaroxaban (10 mg every day) was not associated with any bleeding events.
